# Management of Accidental Succinylcholine Ingestion: Navigating Uncharted Waters

**DOI:** 10.7759/cureus.22312

**Published:** 2022-02-17

**Authors:** Shikha Jain, Pooja Singh, Sunaina T Karna, Zainab Ahmad

**Affiliations:** 1 Department of Anaesthesiology and Critical Care, All India Institute of Medical Sciences, Bhopal, Bhopal, IND

**Keywords:** self-ingestion, depolarizing muscle relaxant, standard operating protocols, psychiatric patient, succinylcholine

## Abstract

Accidental oral ingestion of intravenous drugs is rare and under-reported, which may lead to serious morbidity and worsen the outcome for the patient. Though oral ingestion of sedatives and opioid drugs is reported, literature regarding the ingestion of muscle relaxants and subsequent management is limited. We report an interesting case of oral intake of 10 ml (500 mg) of injection Succinylcholine, a depolarizing muscle relaxant, by a psychiatric patient awaiting electroconvulsive therapy (ECT) in the pre-procedure room. We hereby report the subsequent sequence of events along with the suggested recommendations to be followed in the case of such an eventuality. To the best of our knowledge, this is the first case report of oral ingestion of a depolarizing neuromuscular blocking drug and its subsequent consequences.

## Introduction

Succinylcholine (Sux), a depolarizing neuromuscular blocking agent, is an essential drug in anaesthetic management [1]. Though the preferred route of administration is intravenous, intramuscular and sublingual administration have been reported [2-5]. Although accidental epidural administration of Sux has been reported, there is no literature to guide about the consequences and subsequent management following Sux ingestion.

We report a case of intentional oral intake of Sux by a psychiatric patient awaiting electroconvulsive therapy (ECT) in the pre-procedure room and the subsequent sequence of events along with the suggested recommendations to be followed in case of such an eventuality.

## Case presentation

A 22-year-old man, diagnosed with catatonic schizophrenia, was scheduled for a session of ECT. After informed consent was obtained by the legal representative, the patient was shifted to the pre-operative room under the supervision of the nursing staff, who routinely supervise the patient prior to the procedure. He was compliant with stable vital signs. Without warning, the patient suddenly snatched a vial from the resuscitation emergency cart near his bed, broke it, and drank the contents even as the nursing staff rushed to restrain him. The nursing staff immediately alerted the anaesthesiology team.

The contents of the broken vial were observed to be Sux (50 mg/ml) meant for intravenous injection, which the patient had consumed (10 ml = 500 mg Sux). The patient was assessed for vitals immediately after ingestion and every two minutes thereafter. He was conscious with no respiratory distress. Oxygen was supplemented via a low-flow device. Standard American Society of Anesthesiologists (ASA) monitoring of electrocardiography, non-invasive blood pressure, plethysmography, and peripheral temperature was initiated. No deterioration in oxygenation or disturbance in the vital parameters was noted. After five minutes of Sux ingestion, fasciculations of grade 1 were noted in the lower extremities, which lasted for approximately one minute. No apnoea, jaw rigidity, abnormal heart rhythms, bradycardia, or any change in temperature or end-tidal carbon dioxide (EtCO_2_) was noted. An arterial blood gas sample was unremarkable with a ratio of partial pressure of oxygen to fractional inspired oxygen concentration (PaO_2_/FiO_2_ ratio) of 440, pH 7.43. The serum potassium level was 4.45 mEq/L (normal range 3.5-5.1 mEq/L). The patient was further monitored for the next four hours in a high-dependency unit (HDU). A relative of the patient stayed with him throughout, counseling him and extending emotional support.

A repeat detailed history of any recent trauma, burns, or the presence of any known genetic variations like atypical cholinesterase (which may influence the pharmacokinetics and pharmacodynamics of Sux) was negative. The further course of the hospital stay was uneventful.

## Discussion

Succinylcholine is an ultrashort-acting depolarizing muscle relaxant that works via the stimulation of nicotinic receptors in parasympathetic and sympathetic ganglia [1]. Administration of Sux is reported through intravenous, intramuscular, and submental routes. On intravenous injection, onset occurs in 30 seconds with total paralysis in 45 seconds, while on intramuscular injection, the adequate intubating condition is achieved in three to four minutes [2-4]. Rarely, the use of Sux by sublingual via intraoral or submental routes has also been reported [5]. Paralysis usually proceeds from small, distal, rapidly moving muscles to the proximal, slowly moving muscles. The diaphragm is one of the last muscles to relax. 

There are few cases in the literature reporting accidental epidural injections of Sux. Toleska et al. reported prolonged neuromuscular blockade after epidural administration of Sux [6]. They reported shortness of breath, dysarthria, and fasciculations in the thoracic part of the trunk, upper extremities, and face two minutes after accidental epidural administration of 100 mg of succinylcholine, which was managed with the induction of general anaesthesia. Pourzitaki et al. reported the administration of 125 mg of Sux during combined spinal and epidural anesthesia without any neurological or cardiovascular complications during the postoperative period [7]. They reported that diazepam taken orally can lessen the adverse effects of accidental epidural administration of Sux.

However, even after an extensive literature search, we did not come across any cases of oral administration of Sux, either accidental or intentional, to elucidate the pharmacokinetics and pharmacodynamics of oral administration of this drug. Studies on lactating mothers and breast-fed infants report that Sux is rapidly hydrolysed in maternal plasma (t1/2=3-5 minutes) with very less likelihood of excretion into breast milk or oral absorption in the infants [8,9]. Still, it is unknown if the same applies after ingestion to adult human beings. Though we did note fasciculations after six minutes of ingestion, they were mild, transient, and not associated with any respiratory distress. In spite of the large amount of drug consumed, the absorption of Sux from the oral mucosa may have been minimal due to its polar nature because of its quaternary ammonium composition.

In the absence of any further literature to guide the management of accidental ingestion, we propose the following standard operating protocol (SOP) to be considered for effective management in the case of accidental ingestion of oral succinylcholine (Table 1).

**Table 1:** Standard operating protocol to be followed in case of accidental ingestion of Succinylcholine.

S. No.	Standard operating protocol
1	Reassure the patient and immediately supplement oxygenation with monitoring of heart rate, plethysmography, blood pressure, and temperature.
2	Watch for signs of respiratory insufficiency, establish a patent airway. Assist ventilation if needed. Arrange for a ventilator backup in case patient develops respiratory failure. Monitor and treat, where necessary, for pulmonary oedema.
3	Monitor hemodynamic parameters especially for bradycardia associated with hypotension. Resuscitation with atropine, if needed.
4	Maintain adequate perfusion to all vital organs. Fluid resuscitation and inotropic support should be initiated to target MAP of 75 mmHg, in order to maintain adequate perfusion to all vital organs.
5	Monitor and treat, where necessary, for arrhythmias due to rhabdomyolysis, hyperkalemia, severe metabolic, or respiratory acidosis.
6	Detect early symptoms of malignant hyperthermia including metabolic (elevated CO_2_ production and O_2_ consumption) signs.
7	Neuromuscular monitoring can be performed in case of prolonged apnea to diagnose a phase II block in case of large doses of atypical plasma pseudocholinesterase.
8	Detailed history to rule out the presence of any condition which may affect pharmacokinetics of Sch is a must while resuscitation is being done.

Diagnosis of poisoning or accidental/intentional intake of any drug is even more difficult in a psychiatric patient. Hence, poisoning should be considered in any patient who is exhibiting bizarre behaviour, is subdued/sedated, or who, on clinical examination, has unexplained cardiovascular or respiratory instability. Further, assessment of metabolic parameters like high anion gap or lactate levels may further guide the diagnosis. Some special precautions must be taken to ensure safety within inpatient psychiatric facilities. Safe environments require staff members to be proactive and intervene quickly in tense situations. Their ability to notice and mindfully intervene at these flash-point moments can prevent such untoward events from occurring in the future [10]. Any drug, routine or emergency, must be kept away from the patient’s reach. A proper record must be kept for all drugs with timely checks in each shift so that any intentional misuse/abuse of the drug is noted promptly.

All cases with suspicion of poisoning should be managed as acute medical emergencies using an ABC approach regardless of the agent used. All cases require a focused and detailed history and examination to identify specific physical signs and symptoms of poisoning (Table 2).

**Table 2:** Specific signs and potential treatment in poisoning and overdose. IPPV: intermittent positive pressure ventilation, MAOI: mono amine oxidase inhibitor, SSRI: selective serotonin reuptake inhibitor, TCA: tricyclic antidepressants.

Drug category	Drug	Signs and symptoms	Potential treatments
Anticholinergic	Scopolamine, atropine	Altered mental status, dilated pupils, urinary retention, hyperthermia, dry mucous membranes, Seizures, dysrrhythmias, rhabdomyolysis	Physostigmine; sedation with benzodiazepines, cooling, supportive management
Cholinergic	Organophosphates; carbamates	Salivation, lacrimation, sweating, nausea, vomiting, urination, defaecation, muscle weakness, bronchorrhoea, bradycardia, dilated or constricted pupils, seizures, respiratory failure, paralysis	Airway protection and IPPV, atropine, pralidoxime
Opioid	Heroin, morphine	CNS and respiratory depression, small pupils, Hypothermia, bradycardia, respiratory arrest, acute lung injury	Airway protection, IPPV naloxone
Salicylates	Aspirin	Altered mental status, respiratory alkalosis, metabolic acidosis, tinnitus, hyperpnoea, tachycardia, sweating, low-grade fever, ketonuria, acute lung injury	Alkalinization of urine, potassium (K^+^) repletion, activated charcoal, haemodialysis, hydration
Serotonin syndrome	MAOI, SSRI, TCA	Altered mental status, increased muscle tone, hyperreflexia, hyperthermia, intermittent whole body tremor	Cooling, benzodiazepines
Sympathomimetic	Cocaine; amphetamine	Agitation, dilated pupils, excessive sweating, tachycardia, hypertension, hyperthermia, seizures, rhabdomyolysis, myocardial infarction, cardiac arrest, hyperthermia	Cooling, sedation with benzodiazepines, hydration

Simultaneously, timely use of an antidote may prove to be life saving (Table 3).

**Table 3:** Drugs and their antidotes.

Drug	Antidotes
Benzodiazepines	Flumazenil
Warfarin	Factor II, VII, IX, X concentrate
Digoxin	Digibind
β-Blockers	Glucagon
Acetaminophen	N-acetyl cysteine
Opiate	Naloxone

Since in our case, the ingestion of Sux was witnessed, management was focused on complications reported with Sux administration. However, we did not see any serious adverse events after the ingestion of Sux.

## Conclusions

In conclusion, accidental oral ingestion of Sux can be a problem without complications. However, when there is suspicion of ingestion of anaesthetic agents, serious morbidity can occur and worsen patient outcomes. We suggest that special precautions must be taken to ensure safety within inpatient psychiatric facilities with the availability of proactive staff members with standard operating procedures defined for the management of such emergencies. Any drug, routine or emergency, must be kept away from the patient’s reach. A proper record must be kept for all drugs with timely checks in each shift so that any intentional misuse/abuse of the drug is noted promptly.
